# Prevalence and determinants of anaemia in pregnant women receiving antenatal care at a tertiary referral hospital in Northern Ghana

**DOI:** 10.1186/s12884-019-2644-5

**Published:** 2019-12-11

**Authors:** Anthony Wemakor

**Affiliations:** Department of Nutritional Sciences, School of Allied Health Sciences, University for Development Studies, P O Box TL 1883, Tamale, Ghana

## Abstract

**Background:**

Anaemia during pregnancy is a major public health problem in developing countries. It is important to regularly monitor haemoglobin level in pregnancy and factors associated with it to inform clinical and preventive services. The aim of this study was to assess the prevalence and determinants of anaemia in pregnant women attending antenatal clinic (ANC) of a tertiary referral hospital in Northern Ghana.

**Methods:**

An analytical cross-sectional study involving 400 pregnant women receiving antenatal care in Tamale Teaching Hospital was conducted. Using a semi-structured questionnaire and 24-h dietary recall, data were collected on socio-demographic characteristics, health practices, dietary diversity, anaemia knowledge and haemoglobin level of the women. Anaemia was defined as haemoglobin concentration less than 11 g/dl. Chi-square test and logistic regression analysis were used to identify the independent determinants of pregnancy anaemia.

**Results:**

The mean age and haemoglobin of the women were 28.3 (±4.5) years and 10.81 (±1.41) g/dl respectively. About half of the women 50.8% [95% Confidence Interval (CI): 45.8–55.7] were anaemic and the prevalence of anaemia increased with pregnancy trimester. Among a host of socio-demographic, dietary, and preventive health service factors evaluated, the women’s knowledge on anaemia and pregnancy trimester at interview were the independent determinants of anaemia in pregnancy. Compared to women of the highest anaemia knowledge tertile, women belonging to the lowest (AOR = 2.63, 95% CI: 1.50–4.61) and middle (AOR = 1.92, 95% CI: 1.12–3.27) anaemia knowledge tertiles were about 3 and 2 times more likely to be anaemic respectively. Similarly, women in third trimester of pregnancy were about 4 times more likely to be anaemic compared to those in first trimester at the time of interview (AOR = 3.57, 95% CI: 1.91–6.67).

**Conclusions:**

There is a high prevalence of anaemia, which increases with pregnancy trimester, in pregnant women attending ANC in a tertiary referral hospital in Northern Ghana. The women’s knowledge on anaemia and pregnancy trimester at the time of interview are associated with their anaemia status. The high prevalence of anaemia in pregnancy needs urgent intervention to prevent the occurrence of adverse maternal and neonatal outcomes. Education on anaemia should be intensified at ANCs.

## Background

Anaemia in pregnancy is defined as haemoglobin concentration less than 11.0 g/dl [1]. Globally, anaemia affects half a billion women of reproductive age. In 2011, 29% of non-pregnant women, and 38% of pregnant women aged 15–49 years were anaemic worldwide but the prevalence was highest in South Asia and Central and West Africa [2]. In Ghana, 45% of pregnant women were anaemic in 2014 [3].

Anaemia impairs the capacity of blood to transport oxygen around the body and is an indicator of poor nutrition and health [4]. Anaemia in pregnancy is a major public health issue throughout the world, particularly in the developing countries where it is an important contributor to maternal morbidity and mortality [5]. It is also associated with increased risk of miscarriage [6], prematurity, stillbirth, low birth weight and consequently perinatal mortality [7].

The main cause of anaemia in women of reproductive age globally is iron deficiency, resulting from prolonged negative iron balance, which accounts for 50% of anaemia in women worldwide [2]. The negative iron balance may be due to inadequate dietary iron intake or absorption, increased needs for iron during pregnancy, and increased iron losses as a result of menstruation, worm infestation and infections [8]. Some genetic and socio-demographic and economic characteristics of women also influence the distribution of anaemia [9] and should be taken into consideration in designing preventive interventions for pregnancy anaemia. The World Health Organisation (WHO) recommends intermittent iron and folic acid supplementation for menstruating women living in settings where the prevalence of anaemia is 20% or higher and daily iron and folic acid supplementation for pregnant women as part of antenatal care in order to prevent anaemia in pregnancy [1].

The WHO recommended focused antenatal care approach to antenatal clinic (ANC), which involves client- centered individualized care, disease detection and care by a skilled provider, is implemented in Ghana [10]. In Ghana, all pregnant women are expected to have their haemoglobin measured at first ANC attendance, 28 weeks gestation and 36 weeks gestation to identify and manage anaemia. It is important to assess haemoglobin level of pregnant women and its determinants regularly to inform treatment and preventive services. The objective of this study was to assess the prevalence and determinants of anaemia in pregnant women receiving antenatal care at Tamale Teaching Hospital, a tertiary referral facility in Northern Ghana.

## Methods

### Study design, site, population and subjects

An analytical cross-sectional study involving pregnant women attending ANC of Tamale Teaching Hospital, Northern Region, Ghana, was carried out during February–April, 2016. The Tamale Teaching Hospital is a tertiary level referral hospital for the 4 northern regions i.e., Northern, Savanna, Upper East and Upper West regions. It is located at the north-eastern part of Tamale along the main Tamale-Yendi road in the Tamale South Constituency. The ANC is ran by the maternity department of the hospital. The average daily attendance to the ANC is about 100 women.

Tamale Metropolitan District is located in Northern Region, and has Tamale as its capital. The metropolis has a population size of 371,351 with 274,373 being women and an annual expected number of pregnancies of 4,292 [11]. The people of Tamale Metropolis predominantly belong to the Dagomba tribe; but other minority tribes like Gonja, Konkomba, and Dagarti can also be found in the Metropolis. The main religion of the Metropolis is Islam but a considerable number of people also practice Christian or Traditional African Religion. The Dagombas practise patrilineal lineage system and most men are polygamous.

Pregnant women in Tamale who attended ANC in Tamale Teaching Hospital were the target population of this study.

### Sample size and sampling technique

Sample size was determined using single population proportion formula of Snedecor and Cochran [12]. Using a margin of error (0.05), critical value at 95% confidence level (1.96), and prevalence of anaemia in pregnancy (45.0%) [3], a minimum sample of 380 was estimated. The minimum sample size was increased to 400. About 100 pregnant women attended ANC daily but on each day about 30 pregnant women were sampled using systematic random sampling for 14 days spread over the 3-month study period. For each day a sampling interval of 3 was used. This was obtained by dividing the expected number of women 100 per day by the daily sampling size of 30. One of the numbers 1, 2, and 3 was randomly picked to serve as a starting point and the sampling interval was added to select the next participant until the required number for the day was achieved.

### Data collection

A semi-structured questionnaire and 24-h dietary recall [13] were used to collect the data. The semi-structured questionnaire included sections on socio-demographic characteristics, health practices during pregnancy, dietary practices, knowledge on anaemia in pregnancy and haemoglobin level (Additional file 1). The questionnaire was administered in face-to-face interviews with the women at the ANC.

Seven (7) statements on general nutrition and anaemia were read to the women and they were to indicate whether each statement was true or false. The statements were based on what pregnant women were taught on iron deficiency and anaemia at ANCs in Ghana and were put together by a team of ANC nurses. The statements are: *1. It is very important to eat a variety of foods during pregnancy 2. Good nutrition before conception is not necessary 3. Eating fruits and vegetables (*e.g. *banana and cocoyam leaves) during pregnancy increases the risk of anaemia 4. Iron/folic acid supplements given during ANC are detrimental to blood production in pregnancy 5. Blood loss during pregnancy is normal and hence cannot contribute to anaemia 6. Treating infections (*e.g. *malaria) during pregnancy can reduce the risk of anaemia 7. It is advisable for women that are pregnant to stop eating animal source foods (*e.g. *poultry, fish).*

The most recent results of laboratory tests for haemoglobin, malaria and worm infestation were extracted from the pregnant women’s ANC booklets.

In the 24-h dietary recall, the pregnant women were asked to recall the foods they had consumed in the previous 24 h, first spontaneously then followed by probes to ensure that no meal or snack consumed was left out. The women were then asked to mention all the ingredients of the dishes and snacks they consumed. Using this information, it was indicated whether they ate from the following 10 food groups or not: 1. Grains, white roots and tubers, and plantains, 2. Pulses (beans, peas and lentils), 3. Nuts and seeds, 4. Dairy, 5. Meat, poultry and fish, 6. Eggs, 7. Dark green leafy vegetables, 8. Other vitamin A-rich fruits and vegetables, 9. Other vegetables, and 10. Other fruits [13].

### Study variables

#### Anaemia

Anaemia was defined at haemoglobin concentration less than 11.0 g/dl [4]. Anaemia was further divided into mild (haemoglobin = 10.0–10.9 g/dl), moderate (haemoglobin = 7.0–9.9 g/dl) and severe (haemoglobin < 7.0 g/dl) categories [4].

#### Minimum dietary diversity - women (MDD-W)

Using data from the 24-h dietary recall, dietary diversity score was calculated. For each food group the women ate from they got a score of “1” otherwise a score of “0”. A count of the number of scores was made to give the dietary diversity score [13]. Irrespective of the number of foods eaten from one food group, a score of “1” was given and the total score theoretically ranged from 0 to 10. Using the dietary diversity score, MDD-W was derived. MDD-W is an indicator variable on whether a woman has eaten from 5 or more food groups out of 10 designated food groups within the last 24 h, i.e., a dietary diversity score of 5 or more. MDD-W is a measure of adequacy of micronutrient (including iron) intake among women of reproductive age i.e., women who meet the criterion for MDD-W are less likely to be micronutrient deficient [13].

#### Iron-rich food group

Given that the main variable of interest was anaemia, the iron-rich food group: flesh foods containing organ meat; flesh meat and fish; and sea food, among the 10 food groups was isolated and its association with anaemia was examined.

#### Household wealth index

A household wealth index based on possession of 14 household items was derived. This is based on an earlier concept whereby the sum of dummy variables obtained from data on housing quality, availability of electricity, water and toilet to the household, and ownership of household durable goods (e.g. bicycle, television, radio) and possession of livestock were used to construct a household wealth index [14]. The 14 household items include mattress, TV, satellite dish, animal-drawn cart, mobile phone, radio, sewing machine, car, bicycle, motor bike, computer, electric fan, DVD player and refrigerator. Following exploratory analysis, the percentages of households with mattress, electric fan and mobile phone were above 95% and that of households with animal drawn cart was below 5% so these items were excluded from further analysis. A wealth score was generated for each household using the remaining 10 household items with principal component analysis and the scores for all the households were ordered and divided into 3 categories (tertiles): - lowest, middle and highest.

#### Anaemia knowledge index

Based on the responses to the seven statements on anaemia, a pregnant woman scored “1” when she correctly identified a “true” or “false” statement otherwise “0”. A count of the scores was made to give the total score which could range from 0 to 7 depending on the number of correct answers given by the woman. The total scores were ranked and divided into tertiles i.e., lowest, middle and highest.

### Quality assurance

The questionnaire was first prepared in English, translated into Dagbani and then back translated into English to ensure consistency and accuracy of translation. The questionnaire was also pre-tested on pregnant women seeking antenatal care from another hospital and inconsistencies identified were corrected. A 3-day training was given to data collectors during which they practiced how to ask the questions both in Dagbani and English. Data were collected by 3 final year students of the Department of Nutritional Sciences, University for Development Studies, Tamale. The completed questionnaires were checked for completeness on a daily basis on site and questions that were not answered were answered.

### Data entry and analysis

The data were entered into SPSS then transferred into Stata (version 12) for analysis. Descriptive statistics were computed: frequency and percentage for categorical data, and mean and standard deviation for continuous data. Bivariate and multivariate analyses were employed to identify factors associated with anaemia. Factors that were significant in bivariate analyses with Chi-square or Fisher’s exact test were entered into a multivariate logistic regression model to identify the independent determinants of anaemia in pregnancy. In all statistical tests, a probability value (*p*-value) less than 0.05 was considered statistically significant.

### Ethical consideration

The study received ethics permission from the Joint Ethics Committee of the School of Medicine and Health Sciences and School of Allied Health Sciences, University for Development Studies, Tamale. Written informed consent was obtained from the respondents and they were assured that the information they would provide would be kept confidential and used only for the purposes of the study.

## Results

### Socio-demographic characteristics of respondents

The age of the women ranged from 18 to 41 years with a mean of 28.3 (±4.5) years. Most of the women were in the age group 25–29 years (40.0%), were traders (39.0%), had secondary education (34.5%), and belonged to the middle household wealth index tertile (36.3%) (Table 1). A greater majority of the respondents were married (92.0%), and the majority practiced Islamic religion (61.8%).

**Table 1:** Socio-demographic characteristics of respondents

Characteristic	Frequency (*n* = 400)	Percent (%)
Age group (years)		
< 24	80	20.0
25–29	160	40.0
30–34	123	30.7
35+	37	9.3
Marital status		
Single, never married	15	3.8
Married	368	92.0
Divorced/Separated/Widowed	17	4.3
Educational status		
No education	53	13.3
Primary	11	2.7
Junior High School/Middle School	73	18.2
Senior High School/Vocational School	138	34.5
University	125	31.2
Religion		
Islam	247	61.8
Christian	150	37.5
Others	3	0.7
Occupation		
Trader/Vendor	156	39.0
Agricultural worker	2	0.5
Office worker	40	10.0
Service worker	50	12.5
Education/research	49	12.3
Healthcare	33	8.3
Housewife	70	17.5
Parity		
0	123	30.7
1	99	24.7
2	100	25.0
3	63	15.7
4+	15	3.7
Household wealth index (tertile)		
Lowest	134	33.5
Middle	145	36.3
Highest	121	30.2

### Health status and practices in pregnancy

At the time of interview, 16.3, 41.3 and 42.5% of the women were in first, second and third trimesters of pregnancy respectively, Table 2. About two-thirds of the women (71.0%) initiated ANC in the first trimester. About a third (35.7%) and 9.5% of the women had malaria and worm infestation respectively at one time during the pregnancy, while 2.7% were infected with HIV. Consequently, 25.7 and 9.4% used antimalarial and anti-helminthic drugs during the pregnancy. About a quarter of the women (24.3%) used insecticide treated bed net while a greater majority (98.3%) had used a multivitamin drug containing iron and folic acid. The anaemia knowledge of about a quarter of the women (24.5%) was classified into the highest tertile.

**Table 2:** Health status, preventive health practices and anaemia knowledge of pregnant women

Characteristic	Frequency (*n* = 400)	Percent (%)
Pregnancy trimester at interview		
First	65	16.3
Second	165	41.3
Third	170	42.5
Trimester of first ANC visit		
First	284	71.0
Second	107	26.8
Third	9	2.3
HIV		
No	389	97.3
Yes	11	2.7
Malaria		
No	257	64.3
Yes	143	35.7
Worm infestation		
No	362	90.5
Yes	38	9.5
Antimalaria drug use		
No	292	74.3
Yes	101	25.7
Anti-helmintic drug use		
No	356	90.6
Yes	37	9.4
Bed net use the previous night		
No	303	75.6
Yes	97	24.3
Any multivitamin use		
No	7	1.7
Yes	393	98.3
Anaemia knowledge index (tertile)		
Lowest	140	35.0
Middle	162	40.5
Highest	98	24.5

### Dietary and pica practices

All respondents ate foods from starchy staples (100.0%) whiles only about a fifth ate foods made from eggs (22.8%) (Table 3). A greater majority of the respondents ate flesh foods or foods considered to be rich sources of iron (i.e., meat, poultry, fish) (94.5%) and other vegetables (96.8%) but less than half ate from pulses (beans, peas and lentils) group (43.8%), diary (35.3%), dark green leafy vegetables (44.5%), other vitamin A-rich fruits and vegetables (36.8%) and other fruits (32.8%). However most of the respondents (80.3%) were meeting the minimum dietary diversity of consuming foods from at least five of the ten food groups.

**Table 3:** Food groups women ate from in the past 24 h before the survey and minimum dietary diversity - women

Food group/MDD-Women	Frequency (*n* = 400)	Percentage
Grains, white roots and tubers, and plantain		
Yes	400	100.0
No	0	0.0
Pulses (beans, peas and lentils)		
Yes	175	43.8
No	225	56.3
Nuts and seeds		
Yes	269	67.3
No	131	32.8
Diary		
Yes	141	35.3
No	259	64.8
Meat, poultry and fish		
Yes	378	94.5
No	22	5.5
Eggs		
Yes	91	22.8
No	309	77.3
Dark green leafy vegetables		
Yes	178	44.5
No	222	55.5
Other vitamin A-rich fruits and vegetables		
Yes	147	36.8
No	253	63.3
Other vegetables		
Yes	387	96.8
No	13	3.3
Other fruits		
Yes	131	32.8
No	269	67.3
Minimum Dietary Diversity - Women (MDD-W)		
Yes	321	80.3
No	79	19.8

Eighty-six women practiced some form of pica and 81 of these women started the practice in the first trimester (Table 4). The pica practices involved chewing the following: stick/wooden sponge, cola nuts, clay, chalk and “pepsodent” tooth paste. The main reasons for practicing pica were to prevent nausea and vomiting. Tea/coffee intake was high (75.3%) among the respondents and among those who reported intake, 47.5% took it sometimes and 44.2% took it once a day. The majority (95.8%) of the respondents were not taking any alcoholic beverage during their pregnancy.

**Table 4:** Pica practices of the pregnant women (*n* = 86)

Variable	Frequency	Percentage
Trimester of starting pica		
First	81	94.2
Second	4	4.6
Third	1	1.2
What respondents eat as a form of pica		
Chewing stick/wooden sponge	74	86.0
Cola nuts	15	17.4
Clay	11	12.8
Chalk	7	8.1
Uncooked maize dough/starch	1	1.2
Chewing gum	1	1.2
Pepper	1	1.2
Pepsodent tooth paste	1	1.2
Why respondents practice pica		
To prevent nausea	36	41.9
To prevent vomiting	52	60.2
To prevent salivation	22	25.6
For satiety	4	4.7

### Prevalence of anaemia

The haemoglobin measurements were carried out for most of the women in the second trimester (44.3%), followed by 35.8% in first trimester and 20.0% in third trimester. The mean haemoglobin for the women was 10.81 (±1.41) g/dl and the overall prevalence of anaemia (haemoglobin less than 11.0 g/dl) was 50.8% [95% Confidence Interval (CI): 45.8–55.7]. With respect to the severity of anaemia, 25% each was mildly and moderately anaemic and one woman was severely anaemic. The proportion of anaemic women increased as the pregnancy progressed, with 32.2% in first trimester, 53.7% in second trimester and 77.5% in third trimester.

### Determinants of anaemia

Socio-demographic, socio-economic and anaemia knowledge index of the pregnant women were compared with anaemia status. In the bivariate analyses, pregnancy trimester at interview (*p* < 0.001), religion practiced (*p* = 0.009), household wealth index (*p* = 0.043), trimester of ANC initiation (*p* = 0.010), and anaemia knowledge index (*p* = 0.001) were significant (Table 5). A multivariate analysis including the significant factors in bivariate analyses found the women’s anaemia knowledge index and trimester of pregnancy at the time of interview statistically significant (Table 6). Compared to women of the highest anaemia knowledge tertile, women belonging to the lowest (AOR = 2.63, 95% CI: 1.50–4.61, *p* = 0.001) and medium (AOR = 1.92, 95% CI: 1.12–3.27, *p* = 0.017) anaemia knowledge tertiles were about 3 and 2 times more likely to be anaemic respectively. Similarly, women in third trimester were about 4 times more likely to be anaemic compared to those in first trimester at the time of interview (AOR = 3.57, 95% CI: 1.91–6.67, *p* < 0.001).

**Table 5:** Association between anaemia status and socio-demographic variables

	N	Anaemia status		*P*-value
		Normal (%)	Anaemic (%)	
Age group (years)				
< 24	80	40 (50.0)	40 (50.0)	0.270
25–29	160	84 (52.5)	76 (47.5)	
30–34	123	52 (42.3)	71 (57.7)	
35+	37	21 (56.8)	16 (43.2)	
Marital status				
Married	368	183 (49.7)	185 (50.3)	0.913
Not married^a^	32	14 (43.8)	18 (56.2)	
Educational level				
No education	53	21 (39.6)	32 (60.4)	0.403
Primary	11	6 (54.5)	5 (45.5)	
Junior High School/Middle School	73	35 (47.9)	38 (52.1)	
Senior High School/Vocational School	138	66 (47.8)	72 (52.2)	
Tertiary	125	69 (55.2)	56 (44.8)	
Religion				
Islam	247	109 (44.1)	138 (55.9)	*0.009
Christianity and others	153	88 (58.0)	65 (42.5)	
Occupation				
Trader/vendor	156	70 (44.9)	86 (55.1)	0.585
Agricultural and service worker	52	25 (48.0)	27 (51.9)	
Office worker	40	22 (55.0)	18 (45.0)	
Education/research worker	49	26 (53.1)	23 (46.9)	
Health worker	33	20 (60.6)	13 (39.4)	
Housewife	70	34 (48.6)	36 (51.4)	
Parity				
0	123	64 (52.0)	59 (48.0)	
1	99	51 (51.5)	48 (48.5)	
2	100	47 (47.0)	53 (53.0)	0.709
3+	78	35 (44.9)	43 (55.1)	
Household wealth index (tertile)				
Lowest	134	59 (44.0)	75 (56.0)	*0.043
Medium	145	67 (46.2)	78 (53.8)	
Highest	121	71 (58.7)	50 (41.3)	
Trimester of 1st ANC visit				
First	284	153 (53.9)	131 (46.1)	*0.010^2^
Second	107	42 (39.3)	65 (60.7)	
Third	9	2 (22.2)	7 (77.8)	
Pregnancy trimester at interview				
First	65	44 (67.7)	21 (32.3)	*< 0.001
Second	165	90 (54.6)	75 (45.5)	
Third	170	63 (37.1)	107 (62.9)	
Woman experienced malaria				
Yes	143	64 (44.8)	79 (55.2)	0.180
No	257	133 (51.8)	124 (48.2)	
Woman tested positive for HIV				
Yes	11	5 (45.5)	6 (54.5)	0.798
No	389	192 (49.4)	197 (50.6)	
Minimum Dietary Diversity – Women				
Yes	321	162 (50.5)	159 (49.5)	0.326
No	79	35 (44.3)	44 (55.7)	
Consumption of iron-rich foods				
Yes	378	184 (48.7)	194 (51.3)	0.342
No	22	13 (59.1)	9 (40.9)	
Pica practice				
Yes	86	36 (41.9)	50 (58.1)	0.122
No	314	161 (51.3)	153 (48.7)	
Anaemia knowledge index (tertile)				
Lowest	140	56 (40.0)	84 (60.0)	*0.001
Middle	162	78 (48.2)	84 (51.8)	
Highest	98	63 (64.3)	35 (35.7)	

^2^Fisher’s exact test used for bivariate analysis because of small cell size

^a^Not married includes never married, divorced, widowed, and separated. *Significant at *p*<0.05

**Table 6:** Multivariate analysis of factors associated with anaemia in pregnancy

Variable	Adjusted OR (95% Confidence Interval)	*P*-value
Trimester of pregnancy		
First	1.00	
Second	1.86 (1.00–3.45)	0.051
Third	3.57 (1.91–6.67)	< 0.001
Religion		
Islam	1.00	
Others	0.70 (0.46–1.08)	0.111
Anaemia knowledge index (tertile)		
Lowest	2.63 (1.50–4.61)	0.001
Middle	1.92 (1.12–3.27)	0.017
Highest	1.00	
Household wealth index (tertile)		
Lowest	1.49 (0.88–2.53)	0.135
Middle	1.45 (0.87–2.42)	0.153
Highest	1.00	

## Discussion

The prevalence of anaemia and its determinants in pregnant women attending ANC at a tertiary referral hospital in Northern Ghana were assessed. A high prevalence of anaemia of 50.8% was found among the pregnant women which increased with pregnancy trimester. Following bivariate and multivariate analyses of potential determinants of anaemia, the women’s knowledge on anaemia and pregnancy trimester at interview were identified as the only independent determinants of anaemia.

Most of the study women started ANC visit in the first trimester but the use of insecticide treated bed net was low and consequently a third of them had malaria. Malaria in pregnancy is associated with poor maternal and child health outcomes such as intrauterine growth retardation, preterm birth, low birth weight, and maternal anaemia [15]. To reduce the prevalence of malaria in pregnancy in Ghana, the intermittent preventive treatment of malaria in pregnancy with sulfadoxine-pyrimethamine is to be implemented in all facilities where antenatal care services are provided. However, the level of malaria prevalence measured suggests low sulfadoxine-pyrimethamine uptake among the subjects although that was not assessed in the current study. A fifth of the women practised some form of pica, which has been associated with iron deficiency and anaemia [16], starting from the beginning of the pregnancy. This involved the ingestion of non-food items such as chewing stick/sponge and was aimed at preventing nausea and vomiting. It is important that education on this is intensified in ANCs to prevent unwanted pregnancy outcomes.

A higher prevalence of anaemia (50.8%) was measured than reported for pregnant women in Ghana at 45% in the latest demographic and health survey [3]. Our prevalence rate for anaemia in pregnant women in Northern Ghana is high enough for anaemia to be classified as a severe public health problem according to WHO [17]. In Ghana, our prevalence is lower than reported for groups of pregnant women in 25 communities in Northern Ghana (70.0%) [18], and in Sekyere West District in Southern Ghana (57.1%) [19] but greater than estimated for pregnant women in Sekondi-Takoradi, Western Ghana (34.4%) [8]. In comparison to other pregnancy populations in Africa, our prevalence rate is lower than estimates made for pregnant women in South Eastern Nigeria (58%) [20], Eastern Ethiopia (56.8%) [21], Southern Ethiopia (51.9%) [22], Kiboga, Uganda (63.1%) [23], Derna, Libya (54.6%) [24], and Niger Delta, Nigeria (69.6%) [25]. However, our anaemia in pregnancy prevalence is higher than estimated for populations in South West Ethiopia (23.5%) [26], North West Ethiopia (25.2%) [27], and Mpigi, Uganda (32.5%) [28]. The differences in the prevalence of anemia in pregnancy in the different population groups might be due to differences in socio-economic circumstances, cultural practices, dietary patterns, preventive health practices and diagnostic tests. However, it is clearly established that the prevalence of anaemia in pregnancy is very high and pregnant women are not obtaining enough iron and other essential nutrients from their diet or from supplement use to produce adequate levels of haemoglobin to support pregnancies.

The high prevalence of anaemia measured in our study population may have a number of potential causes including low or non-compliance with iron folic acid supplementation, low dietary iron intake and the high risk nature of the population studied. WHO recommends oral daily iron and folic acid supplementation for pregnant women because of the high iron requirements in pregnancy which cannot be met from dietary sources alone [29]. However, many pregnant women redeem the iron folic acid supplement but do not take it as advised because of the side effects of the elemental iron used in the formulation. In a study, anaemia prevalence increased with non-compliance with iron supplementation as pregnancy progressed and the non-compliant pregnant women were 6 times more likely to be anaemic compared to those compliant (Adjusted OR 6.19, 95% CI 2.55–15.02) [30]. It is also possible that the consumption of iron-rich foods and fruits that aid absorption of iron, zinc and vitamin A needed for hematopoiesis was low in our study population although we found that a high percentage of the women consumed iron-rich foods in the last 24 h before the interviews. Consumption of plant-based foods rich in non-haem iron with low bioavailability underlies this. It is reported that the absorption of non-haem iron may be as low as 5% in plant-based meals with little animal source foods [31]. Women attending ANC of a hospital that receives referrals of pregnant women with complications including anaemia from the four northern regions of Ghana were studied. Thus, it can be assumed that some of the women in the study population could be referred to receive antenatal care at the study hospital because of anaemia-related pregnancy complications but this was not explored in the present study. Infections such as malaria and HIV increase the risk of anaemia [8] and may have also contributed to the level of anaemia measured in the study population.

The study evaluated a host of socio-demographic, dietary, and preventive health service use factors in order to identify risk factors of anaemia for planning health services. Among the potential risk factors identified in bivariate analyses, only the women’s knowledge on anaemia and pregnancy trimester at interview remain significantly associated with their anaemia status in multivariate analysis. Even though religion practised, and household wealth index were significant in bivariate analyses, they did not remain significant in multivariate analysis. The finding of a link between anaemia knowledge and anaemia level is similar to the findings of a survey conducted on pregnant women in Malaysia which found that knowledge on anaemia among the pregnant women corresponds to a reduction in the odds of anaemia [32]. In this study, it is possible that higher levels of anaemia knowledge correspond to more awareness on the causes and preventive strategies of anaemia and this translates into increased adoption of preventive measures hence lower anaemia prevalence rates.

### Limitations

Our study has a number of limitations. One important limitation is that a cross-sectional design, which does not allow for the study of causation, was used. The study did not independently carry out haemoglobin estimation on the women but relied on the hospital’s estimate of haemoglobin of the women recorded in the ANC booklets for anaemia diagnosis. In the assessment of dietary diversity of the pregnant women, only one 24-h dietary recall was conducted, and this may not be representative of the usual dietary pattern individually. This study did not have information on compliance to iron-folic acid supplementation and pregnancy outcomes of the women so could not do any analysis relating to these. Also, the questionnaire used to generate the anaemia knowledge index was investigator-constructed and was not fully validated. Despite these limitations, this study provides some important insights into the level of anaemia in pregnancy and its risk factors in the Tamale Metropolis.

## Conclusions

Half of pregnant women (50.8%) seeking antenatal care at a tertiary referral hospital in Northern Ghana are anaemic making anaemia in pregnancy a severe public health problem. The prevalence of anaemia increased with pregnancy trimester. The women’s knowledge on anaemia and pregnancy trimester at interview emerged as independent determinants of anaemia among a host of socio-demographic, dietary, and preventive service use factors explored. The high level of anaemia in pregnancy needs immediate action to prevent the occurrence of adverse maternal and neonatal outcomes. Education of pregnant women on anaemia should be intensified in ANCs. Further studies are needed to understand why the prevalence of anaemia in pregnancy remains high despite the availability of preventive health services.

## Supplementary information

**Additional file 1.** Study Questionnaire. This is the questionnaire used to collect the study data.

## Data Availability

The datasets used and/or analysed during the current study are available from the corresponding author on reasonable request.

## Abbreviations

ANC: Antenatal Clinic
AOR: Adjusted Odds Ratio
CI: Confidence Interval
g/dl: grammes per deciliter
MDD-W: Minimum Dietary Diversity-Women
WHO: World Health Organisation
