# Supplementary material for: Prevalence and determinants of anaemia in pregnant women receiving antenatal care at a tertiary referral hospital in Northern Ghana
Source: BMC Pregnancy Childbirth. 2019 Dec 11;19:495. doi: 10.1186/s12884-019-2644-5 (PMC6907326; doi:10.1186/s12884-019-2644-5)
Supplement: Supplementary file 1 — Additional file 1. Study Questionnaire. This is the questionnaire used to collect the study data. [file 12884_2019_2644_MOESM1_ESM.docx]

**Additional file 1: Study Questionnaire**

**Prevalence and determinants of anaemia in pregnancy in Northern Ghana**

**(Target group: Pregnant Women Attending ANC at Tamale Teaching Hospital)**

**IDENTIFICATION**

1. Questionnaire No……………..

**SECTION A: SOCIO-DEMOGRAPHIC CHARACTERISTICS OF WOMEN**

**1**. Age of respondent …………………. (years)

**2.** What is your religion?

1. Christianity

2. Islam

3. African Traditional

4. Others (specify): ………………………………..

**3.** Marital status of respondent.

1. Single

2. Married

3. Divorced

4. Widow

5. Separated

6. Others (specify): ……………………………

**4**. Respondents highest educational level completed:

1. None

2. Primary

3. Middle/Junior High School

4. Senior High School/Vocational School

5. Tertiary

6. Others (specify): ………………………………..

**5.** How many children do you have? ……………………….

**SECTION B: SOCIO-ECONOMIC STATUS OF WOMEN**

**1.** Aside from your own housework, what do you do to earn income?

1. Trader/vendor

2. Agricultural worker (e.g. farmer)

3. Office worker (civil servant)

4. Service worker (e.g., hair-dresser, seamstress)

5. Education/research (e.g., teacher)

6. Healthcare (e.g., Nurse)

7. Nothing

8. Others, specify: …………………………………………..

**2.** Does your household have any of these assets? *(Use 1 for Yes and 0 for No)*

| **ASSET** | **RESPONSE** |
| --- | --- |
| Radio |  |
| Color/black TV |  |
| Satellite dish |  |
| Sewing machine |  |
| Mattress |  |
| Refrigerator |  |
| DVD/VCD |  |
| Computer |  |
| Electric fan |  |
| Mobile Telephone |  |
| Bicycle |  |
| Motorcycle/tricycle |  |
| Animal-drawn cart |  |
| Car/truck |  |

**SECTION C: HEALTH PRACTICES IN PREGNANCY**

**1.** Pregnancy trimester of respondent at time of interview.

1. First

2. Second

3. Third

**2.** From the ANC booklet, record the trimester of first ANC visit.

1. First

2. Second

3. Third

**3.** How often do you attend ANC?

1. Monthly

2. Every three months

3. Others (specify): …………………

**4.** Have you had malaria or suspected malaria since conception?

1. Yes

2. No

3. Can’t remember *(Skip to question 6 if response to this question is “No” or “Can’t remember”)*

**5.** Have you had worm infestation since conception?

1. Yes

2. No

3. Can’t remember

**6.** Have you ever tested positive for HIV?

1. Yes

2. No

3. Can’t remember

**7.** Were you given antimalarial medications in this pregnancy?

1. Yes

2. No

**8.** Were you given deworming medications in this pregnancy?

1. Yes

2. No

9. Last night did you sleep under an insecticide treated mosquito net?

1. Yes

2. No

**10**. Record the haemoglobin level of respondent based on the latest estimation from the ANC booklet…………………………… (g/dl).

11. Trimester of this haemoglobin estimation.

1. First

2. Second

3. Third

**SECTION D: DIETARY PRACTICES**

**1.** Do you practice any form of pica?

1. Yes

2. No *(Skip to question 5 if response to this question is “No”)*

2. If yes, what do you eat? *(Tick all that apply)*

1. Chewing stick/wooden sponge

2. Chalk

3. Cola nuts

4. Uncooked maize dough/starch

5. Clay

6. Others

**3.** What stage of your pregnancy did you start this practice?

1. First

2. Second

3. Third

**4.** Why do you practice pica? *(Tick all that apply)*

1 To prevent nausea

2. To prevent vomiting

3. For satiety

4. To prevent salivation

5. Others, specify ________________________________________

**5.** Please do you take any alcoholic beverage?

1. Yes

2. No

**6**. Do you take tea and coffee?

1. Yes

2. No

**7.** If yes, how often do you take it?

1. Once a day

2. Twice a day

3. Weekly

4. Monthly

5. Sometimes

**8.** Do you take some multivitamin supplements?

1. Yes

2. No

**9.** If yes, which of these do you take? *(Tick all that apply)*

1. Folic acid

2. Iron

3. Multivitamin

**10**. Please, mention all the foods and drinks that you ate over the past 24 hours whether at home or outside. (Hint: start with the meal eaten at supper yesterday).

| **Eating moments** | **Name of dish** | **Ingredients** |
| --- | --- | --- |
| Breakfast |  |  |
| Snack before lunch |  |  |
| Lunch |  |  |
| Snack after lunch |  |  |
| Dinner |  |  |
| Snack after dinner |  |  |
| Drinks |  |  |

11. From the meals mentioned by the respondent, indicate whether she ate from the following food groups over the past 24 hours whether at home or outside.

| **Food group** | **Consumed (No=0, Yes=1)** |
| --- | --- |
| Grains, white roots and tubers and plantains |  |
| Pulses (beans, peas and lentils) |  |
| Nuts and seeds |  |
| Dairy |  |
| Meat, poultry and fish |  |
| Eggs |  |
| Dark green leafy vegetables |  |
| Other vitamin A-rich fruits and vegetables |  |
| Other vegetables |  |
| Other fruits |  |

**SECTION E: NUTRITIONAL KNOWLEDGE AND PRACTICE**

*1. Indicate whether each statement below is True or False. Use 1 for “True”, 2 for “False” and 3 for “I don’t know.”*

| **STATEMENT** | **RESPONSE** |
| --- | --- |
| 1. It is much important to eat a variety of foods during pregnancy. |  |
| 1. Good nutrition before conception is not necessary. |  |
| 1. Eating fruits and vegetables during pregnancy increases the chances of being anaemic. |  |
| 1. Iron/folic acid supplements given during ANC are detrimental to blood production. |  |
| 1. Blood loss during pregnancy is normal and hence cannot contribute to anaemia. |  |
| 1. Treating infections (e.g. malaria, diarrhea) during pregnancy can reduce the chance of being anaemic. |  |
| 1. It is advisable for women that are pregnant to stop eating animal source foods (e.g. poultry, fish, meat). |  |
